# Effectiveness of Trigger Point Manual Treatment on the Frequency, Intensity, and Duration of Attacks in Primary Headaches: A Systematic Review and Meta-Analysis of Randomized Controlled Trials

**DOI:** 10.3389/fneur.2018.00254

**Published:** 2018-04-24

**Authors:** Luca Falsiroli Maistrello, Tommaso Geri, Silvia Gianola, Martina Zaninetti, Marco Testa

**Affiliations:** ^1^Department of Neuroscience, Rehabilitation, Ophthalmology, Genetics, Maternal and Child Health, University of Genova, Genoa, Italy; ^2^Unit of Clinical Epidemiology, IRCCS Galeazzi Orthopaedic Institute, Milan, Italy; ^3^Center of Biostatistics for Clinical Epidemiology, School of Medicine and Surgery, University of Milano-Bicocca, Monza, Italy; ^4^UOC di Recupero e Rieducazione Funzionale, Ospedale Policlinico Borgo Roma, Azienda Ospedaliera Universitaria Integrata di Verona, Verona, Italy

**Keywords:** migraine disorders, tension-type headache, cluster headache, trigger points, myofascial pain syndromes, physical therapy specialty

## Abstract

**Background:**

A variety of interventions has been proposed for symptomatology relief in primary headaches. Among these, manual trigger points (TrPs) treatment gains popularity, but its effects have not been investigated yet.

**Objective:**

The aim was to establish the effectiveness of manual TrP compared to minimal active or no active interventions in terms of frequency, intensity, and duration of attacks in adult people with primary headaches.

**Methods:**

We searched MEDLINE, COCHRANE, Web Of Science, and PEDro databases up to November 2017 for randomized controlled trials (RCTs). Two independent reviewers appraised the risk-of-bias (RoB) and the grading of recommendations, assessment, development, and evaluation (GRADE) to evaluate the overall quality of evidence.

**Results:**

Seven RCTs that compared manual treatment vs minimal active intervention were included: 5 focused on tension-type headache (TTH) and 2 on Migraine (MH); 3 out of 7 RCTs had high RoB. Combined TTH and MH results show statistically significant reduction for all outcomes after treatment compared to controls, but the level of evidence was very low. Subgroup analysis showed a statistically significant reduction in attack frequency (no. of attacks per month) after treatment in TTH (MD −3.50; 95% CI from −4.91 to −2.09; 4 RCTs) and in MH (MD −1.92; 95% CI from −3.03 to −0.80; 2 RCTs). Pain intensity (0–100 scale) was reduced in TTH (MD −12.83; 95% CI from −19.49 to −6.17; 4 RCTs) and in MH (MD −13.60; 95% CI from −19.54 to −7.66; 2RCTs). Duration of attacks (hours) was reduced in TTH (MD −0.51; 95% CI from −0.97 to −0.04; 2 RCTs) and in MH (MD −10.68; 95% CI from −14.41 to −6.95; 1 RCT).

**Conclusion:**

Manual TrPs treatment of head and neck muscles may reduce frequency, intensity, and duration of attacks in TTH and MH, but the quality of evidence according to GRADE approach was very low for the presence of few studies, high RoB, and imprecision of results.

## Introduction

Headaches are disabling disorders that decrease the health-related quality of life. The mean global annual prevalence of headaches in adults is around 46% (1), while in Europe the gender adjusted 1-year prevalence for any type of headache is 79% (2) with an estimated economic burden of €173 billion yearly among adults aged 18–65 years (3). As in other European countries, headaches in Italy are highly prevalent and associated with significant personal impact and important implications for health policy (4). Therapeutic management option is represented by pharmacological or non-pharmacological interventions (5). Pharmacological treatments are considered the main interventions even though the elevated frequency of attacks increases the risk of drugs’ abuse (6). Accordingly, the use of non-pharmacological treatments in the management of primary headaches has the aim of reducing the drugs’ consumption, their side effects, and the interaction with the other drugs used for comorbidities (7–9). The recommendation of the European Federation of Neurological Societies guideline indicates that the use of non-pharmacologic therapies, having less side effects than pharmacological therapies (10, 11) may constitute a valid therapeutic option for headaches sufferers, despite their limited scientific basis (12), and the lack of research on the effectiveness of physical therapy treatments.

Tension-type (TTH), migraine (MH), and cluster headache (CH) are considered the three most prevalent primary headaches according to the classification proposed by the International Headache Society (13) that established the clinical criteria useful in the discrimination of headache’s attacks. Hence, distinct and specific etiopathological models have been proposed for TTH (14, 15) and MH (16) despite this is a still disputed aspect. In fact, although the IHS classification has been recognized as the most important of the past years (17) some patients with headache may present with overlapping clinical features that makes it difficult to correctly classify them, especially in the case of TTH and MH (18). The concept of a continuum spectrum between these disorders has been proposed to overcome this taxonomic problem (19) and since, its validity has been debated in the past decades (20–22) as the clinical characteristics of patients in both TTH and MH groups come across with more similarities than differences. Patients may share demographic characteristics for specific age groups and respond positively to similar treatments (23); also most triggering factors (e.g., stress) are shared between MH and TTH (24, 25). Moreover, alterations like decreased gray matter in brain areas associated with pain transmission (anterior cingulate, insular cortices, and the dorsal rostral pons) are common in patients with TTH and MH (26, 27). In the so-called continuum model, MH and TTH represent different points on a single continuum of severity, with migraine falling at the more severe end of the symptom spectrum (28–30). Therefore, the different clinical headache expressions are considered the result of the different extent of sensitization occurring at the first- and second-order neurons of the trigeminal nucleus caudalis (TNC) induced by alterations of the trigeminal nerve (30).

Despite the neurophysiologic influence of trigger points (TrPs) on pain mechanisms seems attributable to their action as nociceptive sources (31, 32), the pathophysiology of TrPs on pain still needs to be elucidated (33, 34). Patients with chronic TTH and MH have a greater number of TrPs compared to healthy subjects (35–37); the higher number of TrPs correlates with the severity and the duration of TTH attacks (38), but not in MH attacks (36). Furthermore, even though the pain of MH is predominantly associated with the activation of the trigeminal-vascular system (16), TrPs can be seen as additional stimuli that may contribute to start a migraine attack (37, 39, 40) and the inhibition of TrPs by mean of anesthetic injections led to a decrease in the frequency and severity of migraine attacks (41). The same intervention also promoted the reduction of attacks’ frequency and intensity in a case series of patients with CH (42). However, it is unclear whether the TrPs may have a pathogenetic role (14) or constitute a precipitating factor of separate clinical conditions (15). In both cases, the TrPs are involved in the pathophysiology of primary headaches as a peripheral mechanism able to sensitize the TNC (43, 44). Whatever the influence of TrPs on the pathophysiology of TrPS will be, these findings suggest that the TrPs treatment could be useful to prevent or decrease the extent of primary headaches.

Myofascial TrPs treatment is usually pursued with invasive (dry or wet needling) or non-invasive techniques (manual treatment or low-level laser therapy) (32) that, according to the most accepted hypothesis (33), are thought to reduce the ischemia-related nociception activated by the contracture of a small portion of muscle and, consequently, the degree of sensitization of TNC. Among the manual treatment of TrPs several techniques have been proposed that act directly or indirectly on the TrPs. Techniques that are thought to reduce the muscle contraction with mechanical forces (compression, distraction) acting directly on the TrPs site or the surrounding tissues are ischemic compression (45), myofascial release (46), acupressure (47), and specific soft tissues mobilization techniques (48). Indirect techniques, such as muscle energy (49), positional release (50), and strain–counterstrain techniques (51) are thought to reduce muscle contraction for neurophysiological mechanisms regulating the muscle tone.

Several reviews have considered the efficacy of various interventions used in physiotherapy to treat primary headaches, including many manual therapies, multimodal and manipulative approaches, but the results were usually obtained grouping heterogeneous or combined treatment and considering primary headaches as separate entities (5, 9, 52–56). To our knowledge, the effectiveness of neither manual TrPs treatment in case of primary headaches has not been investigated yet nor it has been established considering patients with different primary headaches (MH, TTH) an homogeneous patient group, as proposed by the continuum severity model. The main purpose of this review was to establish the effectiveness of manual treatment of TrPs in reducing frequency, intensity, and duration of primary headaches. We will also investigate additional positive or negative effects of the manual treatment of TrPs and whether there is a manual technique or a treatment dosage to prefer.

## Methods

We conducted this systematic review following the preferred reporting items for systematic reviews and meta-analyses statement (57) and the Cochrane handbook for systematic reviews of interventions (58). The protocol of the review was registered in PROSPERO, an international prospective register of systematic reviews (registration code CRD42016046374) (59).

### Eligibility Criteria

#### Types of Studies

Randomized controlled trials (RCTs) written in English, Italian, or Spanish.

#### Types of Participants

Participants included in the studies were adult subjects (age >18 years) with primary headaches (TTH, MH, CH) diagnosed using the International Classification of Headache Disorders criteria (13).

#### Types of Interventions

Any direct or indirect manual treatment targeting the TrPs, such as compression techniques, muscle energy techniques, myofascial techniques, acupressure, soft tissues techniques, or positional release techniques. Studies that proposed different approaches were considered only if the manual TrPs treatment was proposed as the only treatment in one of the intervention groups (60). Studies without manual TrPs treatment or referring to any pharmacological or injection treatment, spinal manipulation, and exercise without manual TrPs treatment were excluded.

#### Types of Comparators

Acceptable comparators were any type of minimal active intervention (placebo, sham treatment, routine medication, or wait list supported with routine medication) or no active intervention (wait list or no treatment).

#### Types of Outcomes

We considered as primary outcomes the variation of frequency of the attacks (number of attacks per month), the intensity of attacks [valuated with the 0–100 mm visual analog scale (VAS) or 0–10 numerical pain intensity scale—NPRS or similar scales], and duration of attacks (number of hours per attack). Secondarily, we also considered quality of life, medicine consumption, effects on TrPs, and any adverse event, such as dizziness, bruising, muscle stiffness, and post-treatment pain. Each outcome was considered in the period after the intervention (run-out period).

### Search Strategy

The literature search was performed by two independent authors (FML and ZM) on MEDLINE, COCHRANE, Web Of Science, and PEDro databases, without the adoption of limits or filters. Relevant reviews were consulted for additional studies to consider. Last search was done on November 17, 2017. The full search strategy through all databases is reported in Appendix S1 in Supplementary Material.

### Study Selection

Two independent authors (FML and ZM) screened the record by title and abstract applying the eligibility criteria. At the end of the screening process, full-text articles were retrieved and assessed for their eligibility in the qualitative and/or quantitative synthesis. Any disagreement was resolved by consensus, otherwise a third author (GT) made the final choice. The inter-reviewers agreement of the eligibility process before consensus was expressed using the Cohen’s kappa.

### Data Collection

Two authors (FML and ZM) independently collected information from the included trials by an *ad hoc* extraction form. The extracted data were inserted into a pre-formatted table for studies’ characteristics, such as author, year, design, country, headache diagnosis, sample size calculation, number of participants recruited, drop-outs, intervention (treatment and control), duration, follow-up, outcomes, and measure unit. Disagreements between reviewers were resolved by consensus or if no agreement could be reached, a third author (GT) was consulted.

### Risk of Bias (RoB) Assessment

The internal validity on the included studies was assessed with the Cochrane RoB tool (58). The following domains were appraised: selection bias (random sequence generation and allocation concealment), performance bias (blinding of participants and personnel), detection bias (blinding of outcome assessment), attrition bias (incomplete outcome data), and reporting bias (selective reporting) (58). Each domain could be classified as “high,” “low,” or “unclear” RoB if the study did not provide sufficient information to judge. Two reviewers (FML and ZM) independently assessed the above-mentioned domains of RoB. Any disagreement was resolved by consensus or if no consensus was obtained a third reviewer (GT) made the final choice. Strength of inter-raters agreement before consensus was expressed using the Cohen’s kappa.

### Analysis and Synthesis of Results

We evaluated the treatment effects for dichotomized outcomes using the risk ratio (RR), and for continuous outcomes using the pooled mean difference (MD). The variance was expressed with 95% confidence intervals (95% CI). The outcome measures from the individual trials were combined through meta-analysis were possible using random-effects models described by DerSimonian and Laird (61) because a certain degree of heterogeneity of population and treatments would be expected among interventions of trials. If any planned outcome was not reported quantitatively, a comprehensive description was reported. Since similar scales with different ranges were used to measure the intensity of pain (e.g., VAS 0–10 and VAS 0–100), we linearly transformed each scale into a 0–100 scale (correcting the SDs accordingly) in order to directly compare the pain scales. This method proved to be suitable for being able to directly compare different pain scales (62).

For the meta-analyses, we entered the mean values and SDs measured after the intervention (run-out). If SDs were not reported, we calculated it from SEs. All frequencies of attacks were normalized in attacks per month (mean and SD).

The units of randomization and analysis in the included trials were the individual participants. When a trial presented multiple comparisons, in order to overcome a unit-of-analysis error we used a suggested method (63) that consisted in splitting the participants of the intervention (64) or the comparison groups (65) in two or more groups with smaller sample size, but equal means and SDs in order to avoid the loss of data that could have occurred when only a single pair of interventions is chosen or a treatment group (TG) is deleted.

We analyzed heterogeneity by means of the *I*^2^ statistic and the Chi^2^ test. A *P*-value of less than 0.1 indicated a statistically significant heterogeneity for the Chi^2^ test (66). The percentage of *I*^2^ represented the degree of heterogeneity: percentages of 25, 50, and 75% indicated a low, moderate, and high degree of heterogeneity, respectively (66). If a study did not provide usable summary measures for an outcome it was included in the review, but excluded from the meta-analysis, e.g., Lawler and Cameron (67). For included studies, the number of lost to follow-up in each group and reasons for attrition were recorded. For missing data, similarity of group was evaluated, then the corresponding authors of included studies were contacted (e.g., emailing or writing to corresponding author/s) and if no information were provided, we conducted analyses using only available data (i.e., we did not impute missing data) (63).

We pooled studies through a subgroup analysis according to the type of specific headache. Furthermore, sensitivity analyses for RoB assessment were planned in case of sufficient numbers of studies in each pairwise comparison.

The software used was Cochrane Review Manager Version 5.3 (68).

### Level of Evidence

To evaluate the overall quality of evidence, we used the grading of recommendations assessment, development and evaluation approach (GRADE), for the main outcome based on the methodological quality of included trials (69). The highest quality rating is for evidence based on RCTs with a low RoB. However, it is possible for authors to downgrade this level of evidence to moderate, low, or even very low. The quality of evidence depends on the presence of five factors: study limitation (RoB), indirectness of evidence, unexplained heterogeneity or inconsistency of results, imprecision of results, and high probability of publication bias (70). For the RoB factor, the evidence was arbitrary downgraded by one level when two criteria were judged as high or unclear, and by two levels when more than two criteria displayed high or unclear risk. The software used was GRADEPro GDT (71).

Two authors (FML and ZM) independently assessed the quality of evidence and the RoB. In case of doubt, another reviewer (GT) was consulted for the final choice. Strength of inter-raters agreement before consensus was expressed using the Cohen’s kappa.

## Results

### Study Selection

**Figure 1:**
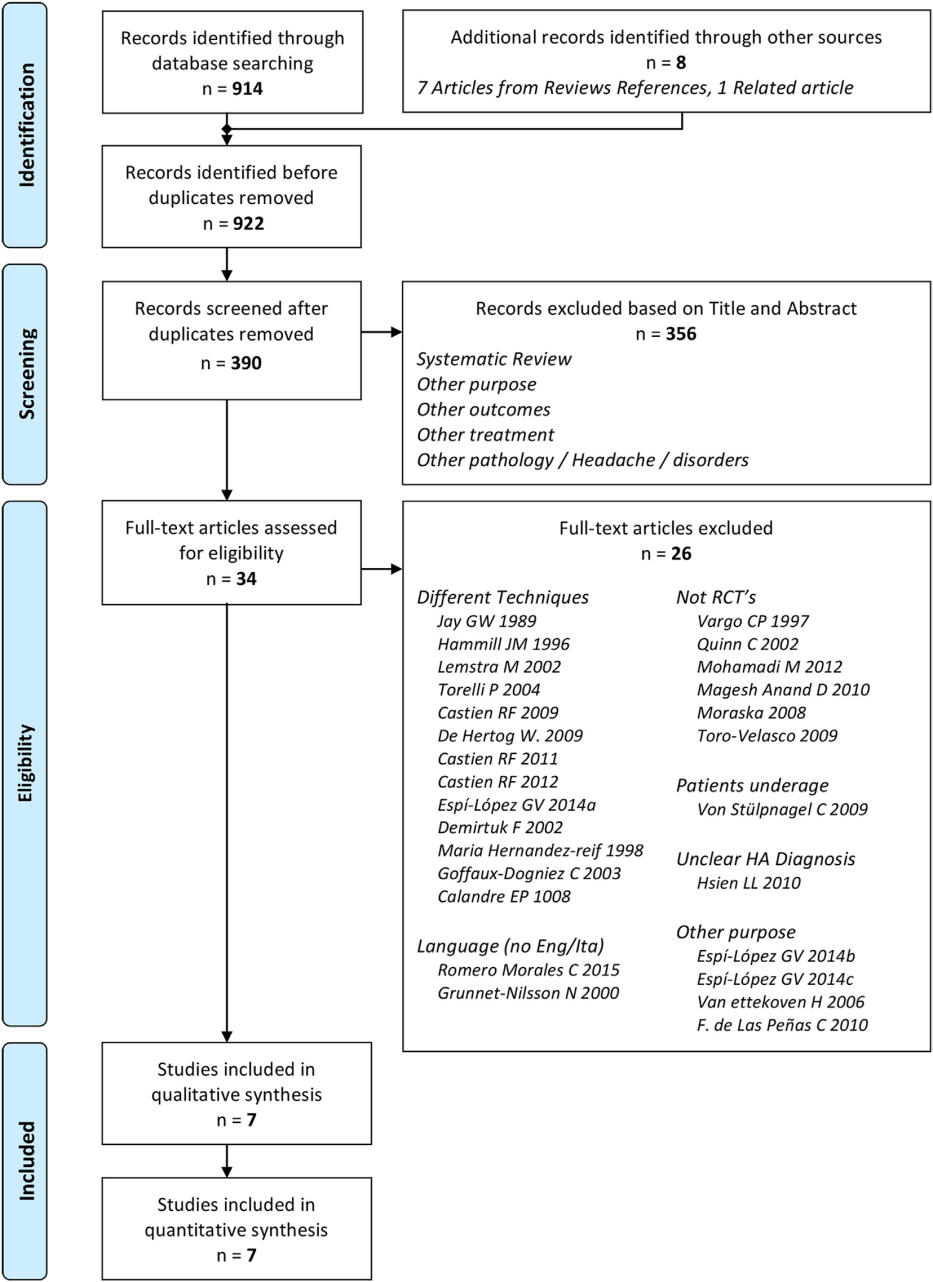
Studies selection flow diagram.

We identified 914 records through databases searching and 8 from additional references (52, 53, 72, 73). After removal of duplicates, a total of 390 records were selected for screening. We excluded 356 records after reading title and abstract. Of the 34 remaining articles assessed, only seven were considered eligible (Figure 1) (60, 64, 65, 67, 74–76). The inter-reviewer agreement regarding study eligibility was moderate with a kappa of 0.69.

### General Study Characteristics

All studies were RCTs; five trials concerned TTH (60, 64, 65, 74, 75) and two MH (67, 76). No studies were found concerning CH (Table 1).

**Table 1:** Characteristics of included studies.

Reference, Design, Country	HA diagnosis, No. of subjects recruited, drop-out, sample size calculation (primary outcome), trial arms, and No. of participants	Intervention treatment/control	Duration/follow-up	Outcomes/measure units
Ajimsha (65), RCT, India	CTTH/ETTH (diagnosis by neurologist) *N* = 63 (36 F, CTTH = 9, ETTH = 47) Drop-out: 7 Sample size calculation: no Trial arm: *N* (mean age ± SD) TG1: 22 (43.7 ± 5.6) TG2: 22 (44.7 ± 5.2) CG: 12 (43.0 ± 5.4)	All groups: two 60′ sessions per week for 12 weeks. TG1: treatment with DT-MFR protocol. TG2: treatment with IDT-MFR protocol. CG: slow soft stroking with finger pads all over the head in the same area of MFR techniques.	Duration 16 weeks: – 4 weeks baseline – 12 weeks treatment Run-out: – 4 weeks post treatment	Self reported daily headache diary – Frequency: no. of days with HA per 4 weeks – Intensity and duration: not assessed

Berggreen (75), RCT, Denmark	CTTH (diagnosis by general practitioner) *N* = 43 (43 F) Drop-out: 4 Sample size calculation: yes (pain) Trial arm: *N* (mean age ± SD) TG: 19 (38.8 ± 13.7) CG: 16 (42.3 ± 10.2)	TG: one session of TrPs treatment with ischemic compression per week for 10 weeks. CG: no treatment	Duration 14 weeks: – 4 weeks baseline – 10 weeks treatment Run out: – 4 weeks post treatment	Self reported daily headache diary – Intensity: 100 mm VAS – Frequency and duration: not assessed Other outcomes: – Inconvenience of the pain: 100 mm VAS – Medicine consumption – Number of TrPs – Multidimensional pain: MPQ Mc gill pain questionnaire; – Quality of life: SF36

Ferragut-Garcías (60), RCT, Balearic Islands	CTTH/FETTH (diagnosis by neurologist) *N* = 100 (78 F, CTTH = 41, FETTH = 56) Drop-out: 3 Sample size calculation: yes (HIT-6) Trial arm: *N* (mean age ± SD) TG1: 23 (38.1 ± 10.9) TG2: 25 (39.4 ± 11.0) TG3: 25 (40.8 ± 12.1) PG: 24 (40.5 ± 12.0)	All groups: Six 15′ sessions: 2 in the first week, 2 in the second week, and 1 each in the third and the fourth week. TG1: 3′ of soft tissue techniques in each pair of craniocervical region muscles. TG2^a^: neural mobilization techniques TG3^a^: treatment 1 and 2 combined PG: sham massage	Duration 6 weeks: – 2 weeks baseline – 4 weeks treatment Run out: – 2 weeks post treatment Follow-up: – 4 weeks after treatment	Self reported daily headache diary – Frequency of attack: no. of days with HA in 2 weeks – Intensity: 0–10 mm VAS – Duration: not assessed Other outcomes: – Pressure pain treshold – Quality of life: headache impact test-6 HIT-6

Ghanbari (74), RCT, Iran	CTTH (diagnosis by neurologist) *N* = 30 (28 F) No drop-out. Sample size calculation: estimated from previous study; primary outcome not spec. Trial arm: *N* (mean age ± SD) TG: 15 (37.7 ± 8.6) CG: 15 (36.3 ± 7.5)	TG: five sessions (from 60′ to 100′) of positional release therapy (PRT) in 2 weeks. CG: routine medications with NSAIDs as abortive drugs and TCAs as prophilactic drugs	Duration 4 weeks: – 2 weeks baseline – 2 weeks treatment Run-out: – 2 weeks post treatment	Self reported daily headache diary – Frequency of attack: no. of days with HA in 2 weeks – Intensity: 10 point NPI – Duration: number of hours per attack. Other outcomes: – Tablet count: no. of pain rescue tablet used – TrPs sensitivity measured by digital force gage and numeric pain intensity

Ghanbari (76), RCT, Iran	MH (diagnosis by neurologist) *N* = 44 (24 F) No drop-out Sample size calculation: no Trial arm: *N* (mean age) TG: 22 (38.7) CG: 22 (35.9)	TG: five 90′ sessions of PRT in 2 weeks + routine medications with NSAIDs, nortriptyline, propranolol, and depakine. CG: routine medications with NSAIDs, nortriptyline, propranolol and depakine	Duration 4 weeks: – 2 weeks baseline – 2 weeks treatment Run out: – 4 weeks post treatment Follow-up: – 2 and 4 months post treatment	Self reported daily headache diary – Frequency of attack: no. of days with HA – Intensity: 0–5 pain scale – Duration: number of hours per attack. Other outcomes: – Tablet count: no. of pain rescue tablet used – TrPs sensitivity measured by digital force gage – Cervical range of motion by clinical goniometer (flexion, extension, rotation, lateral flection)

Lawler (67), RCT, New Zealand	MH (diagnosis by questionnaire) *N* = 48 (40 F) Drop-out: 4 Sample size calculation: no Trial arm: *N* (mean age ± SD) GT: 23 (41.3 ± 13.5) GC: 21 (41.3 ± 13.5)	GT: one 45′ sessions every week for 6 weeks. Trigger-point treatment of the back, shoulders, neck, and head with myofascial release (3 min), deep ischemic compression, and cross-fiber work CG: no treatment	Duration 13 weeks: – 4 wk baseline – 6 wk treatment. Run out: – 3 wk post treatment	Self reported daily headache diary – Frequency of attack: no. of days with HA – Intensity: 0–5 pain scale – Duration: not assessed. Other outcomes: – Drugs consumption – Effect after treatment on heart rate, state anxiety, and salivary cortisol. – Stress and coping: perceived stress scale. – State anxiety: STAI-sf state anxiety scale

Moraska (64), RCT, USA	CTTH/FETTH (diagnosis by neurologist) *N* = 62 (48 F, CTTH = 30, FETTH = 26) Drop-out: 6 Sample size calculation: yes (frequency) Trial arm: *N* (mean age ± SD) TG: 17 (32.1 ± 12.0) PG: 19 (34.7 ± 11.1) CG: 20 (33.4 ± 9.0)	All groups: two 45′ session per week for 6 weeks. TG: manual TrPs treatment composed by 15′ of myofascial release, 20′ of trigger point release massage TRP, 10′ PIR PG: detuned ultrasound in specified head and neck muscle areas CG: no treatment	Duration 10 weeks: – 4 wk baseline – 6 wk treatment Run out: – 4 wk post treatment	Self reported daily headache diary – Frequency of attack: no. of days with HA in 4 weeks – Intensity: 100 mm VAS – Duration: number of hours per attack. Other outcomes: – Use of pain medication – Perceived clinical change – Pressure pain treshold – Quality of life: headache disability inventory and headache impact test-6 HIT-6

*RCT, randomized controlled trial; F, female; wk, weeks; CTTH, chronic tension type headache; ETTH, episodic tension type headache; FETTH, frequent episodic tension type headache; MH, migraine; TG, treatment group; CG, control group; PG, placebo group; VAS, visual analog scale; NPI, numeric pain index; HA, headache; wk, weeks; TrPs, trigger points*.

*^a^Results from these groups were not included into quantitative analysis*.

The pooled population was of 316 subjects (mean age 39.0 ± 11.6 years) with a significant majority of female subjects (251 F, *P* < 0.05). A mean of 45.1 (±9.2) patients were randomized in each trial. One RCT concerned chronic TTH (75, 77), one concerned episodic TTH (65), two considered both frequent-episodic TTH and chronic TTH (60, 64), one RCT concerned MH with and without Aura (67), and two RCTs concerned undefined/unclassified TTH (74) and MH (76).

The TTH subgroup consisted of 225 subjects (71% of the whole population; mean age 38.9 ± 9.8 years) with a significant majority of female subjects (188 F, *P* < 0.05). One hundred and twenty-six subjects had chronic TTH (60, 64, 74, 75, 77), 55 had frequent-episodic TTH (60, 64), and 47 had episodic TTH (65).

The MH subgroup consisted of 91 subjects (29% of the whole population; mean age 39.3 ± 13.5 years) with a not significant majority of female subjects (63 F, *P* = 0.41). Sixty subjects had MH without Aura (67, 76) and 31 had MH with Aura (67, 76).

Headaches were diagnosed using the International Classification of Headache Disorders criteria (13). In five trials (60, 64, 65, 74, 76), the diagnosis was done by a neurologist while in two trials the diagnosis was made either on the score of the Headache History Inventory (67) or by a general practitioner (75).

All the selected studies compared manual treatment versus minimal active treatment: placebo (64), sham massage (60, 65), routine medication supported with pharmacological prophilactic treatment (74), and wait list supported with routine medication (64, 67, 75, 76).

### Treatment and Sessions

The duration of intervention and the number of treatments ranged from 5 sessions in 2 weeks to 24 sessions in 12 weeks with an average number of 2 sessions per week; the run-out/follow-up period varied from a minimum of 2 weeks to a maximum of 4 months after the end of treatment. The duration of the single session ranged from 15 to 100′.

The treatments proposed across all studies were heterogeneous: ischemic compression, myofascial release, muscle energy, soft tissue treatment, and positional release (Table 2).

**Table 2:** Classification of the different TrPs manual treatment techniques.

Reference	Headache	Compression techniques	Muscle energy techniques	Myofascial techniques	Soft tissues techniques	Positional release techniques
				
		Ischemic compression Pressure release TRP Trigger Point Release Massage	PIR CRAC	Myofascial release DT/IDT-MFR	SSTM Deep transverse friction	PRT
Ajimsha (65)	TTH			X		
Berggreen (75)	TTH	X				
Ferragut-Garcías (60)	TTH	X				
Ghanbari (74)	TTH					X
Moraska (64)	TTH	X	X	X		
Ghanbari (76)	MH					X
Lawler (67)	MH	X		X	X	

*TTH, tension type headache; MH, migraine; PIR, post isometric relaxation; CRAC, contract relax antagonist contraction; DT/IDT-MFR, direct/indirect myofascial release; SSTM, specific soft tissue mobilization; PRT, positional release therapy*.

Stretching was often used as warm-up and cool-down technique. The muscles mainly treated were the upper trapezius, the sternocleidomastoid, and the suboccipitals; secondarily, other neck or head muscles were treated (Table 3).

**Table 3:** Muscles and/or muscle groups treated in the trials.

Reference	Headache	Upper trapezius	Suboccipital	Sternocleidomastoid	Masseter	Temporalis	Pterygoid medial/lateral	Anterior neck muscles	Facial	Splenius Capitis	Splenius Cervicis	Occipital	Posterior Cervical	Cervical Multifidus	Rotators-Interspinalis	Levator Scapulae	Semispinalis Capitis
Ajimsha (65)	TTH	X	X	X									X				
Berggreen (75)	TTH	X	X	X	X	X	X	X	X	X	X	X	X				
Ferragut-Garcías (60)	TTH	X	X	X	X	X											
Ghanbari (74)	TTH	X	X	X										X	X		
Moraska (64)	TTH	X	X	X													
Ghanbari (76)	MH	X	X	X										X	X		
Lawler (67)	MH	X	X	X	X	X										X	

*TTH, tension type headache; MH, migraine*.

### RoB Within Studies

**Figure 2:**
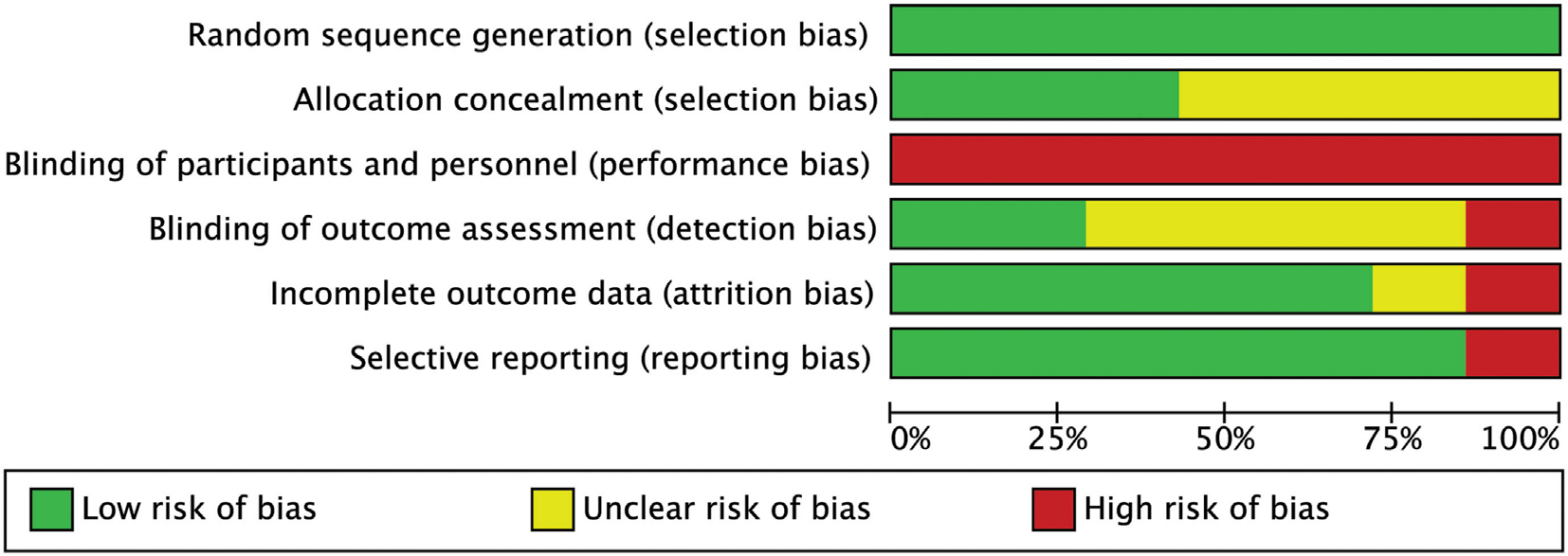
Risk of bias (RoB) graph: review authors’ judgments about each RoB item presented as percentages across all included studies.

**Figure 3:**
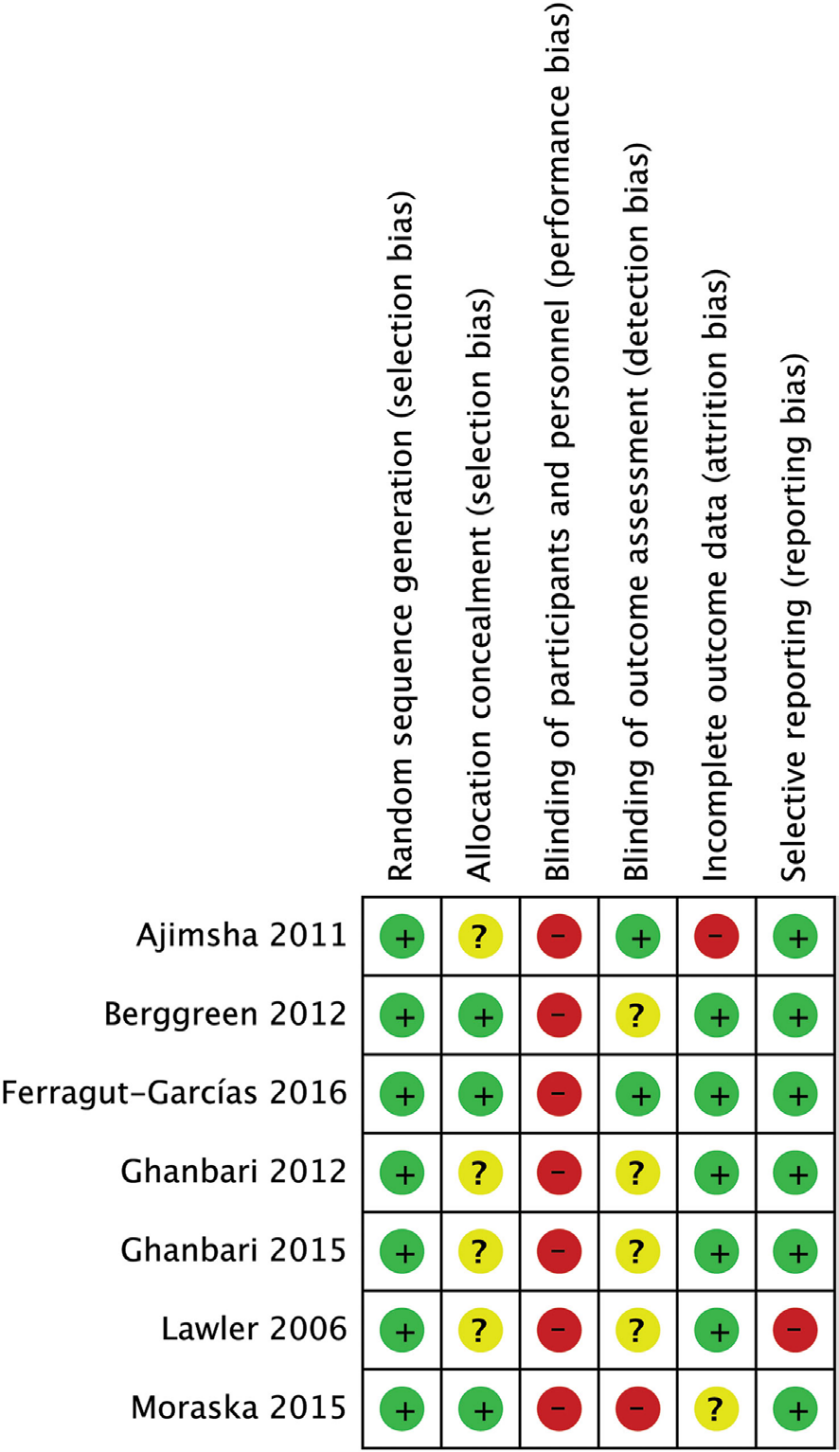
Risk of bias (RoB) summary: review authors’ judgments about each RoB item for each included study.

None of the studies had a low RoB for all methodological items, while 3 out of 7 studies had more than one high RoB domain (64, 65, 67). Three RCTs had low risk in allocation concealment (60, 64, 75). As we expected for manual interventions, the blinding of participants and providers was always unachievable while blinding of assessors was adequately reported only in 2 out of 7 studies (60, 65). One RCT had high risk for incomplete outcome data because some patients did not maintain headache diaries as advised (65). One RCT had high risk in selective reporting because primary data about intensity were not provided (67) (Figures 2 and 3). The agreement between reviewers for individual domains of the Cochrane RoB tool was moderate (*k* = 0.72). Consensus was always achieved between the pair of initial reviewers.

### Synthesis of the Results

For primary outcome (frequency, intensity, and duration), the original measures extracted in each trial are reported in Table 4.

**Table 4:** Original values extracted from trials regarding frequency, intensity, and duration in TTH and MH measured at baseline, after treatment, and at follow-up.

Outcome	Study	Headache	Comparator	Scale	Group	Randomized	Drop out	Available	Baseline	Post treatment/run-out	Follow-up
Frequency of attacks	Ferragut-Garcías (60)	TTH	Sham massage	Attacks × 4 weeks^a^	Treatment	25	2	23	**17.2 ± 4.6**	**9.4 ± 2.8**	**9.6 ± 3.4**
					Control (sham massage)	25	1	24	**14.4 ± 5.4**	**13.8 ± 5.0**	**13.6 ± 4.6**
	Moraska (64)	TTH	(1)Placebo	Attacks × 4 weeks^a^	Treatment	20	3	17	**14.88 ± 0.92**	**10.44 ± 1.3**	
			(2)Wait list		Placebo (detuned US)	21	2	19	**15.24 ± 0.84**	**11.84 ± 1.31**	
					Control (wait list)	21	1	20	**14.76 ± 0.84**	**12.86 ± 1.27**	
	Ajimsha (65)	TTH	Sham massage	Attacks × 4 weeks	Treatment 1	25	3	22	12.0 ± 2.8	4.9 ± 1.7	
					Treatment 2	25	3	22	12.4 ± 2.8	5.7 ± 1.3	
					Control	13	1	12	12.0 ± 2.5	10.4 ± 2.7	
	Ghanbari (74)	TTH	Routine medication	Attacks × 4 weeks^a^	Treatment	15	0	15	**24.88 ± 4.44**	**15.32 ± 8.90**	
					Control	15	0	15	**22.26 ± 3.98**	**20.40 ± 4.80**	
	Lawler (67)	MH	Wait list	Attacks × 4 weeks^a^	Treatment	24	1	23	**6.08 ± 5.00**	**4.28 ± 5.36**	
					Control	24	3	21	**7.04 ± 4.96**	**6.88 ± 5.32**	
	Ghanbari (76)	MH	Routine medication	Attacks × 4 weeks^a^	Treatment	22	0	22	**8.00 ± 2.54**	**4.26 ± 1.64**	**4.62 ± 1.98**
					Control	22	0	22	**7.9 ± 1.98**	**6.08 ± 2.34**	**5.62 ± 1.90**

Intensity of attacks	Ferragut-Garcías (60)	TTH	Sham massage	0–10 VAS^b^	Treatment	25	2	23	**44 ± 11**	**28 ± 8**	**28 ± 10**
					Control	25	1	24	**56 ± 11**	**54 ± 10**	**54 ± 11**
	Moraska (64)	TTH	(1)placebo	0–100 VAS	Treatment	20	3	17	31.4 ± 2.69	22.8 ± 3.03	
			(2)Wait list		Placebo (detuned US)	21	2	19	33.3 ± 2.52	31.5 ± 2.80	
					Control (wait list)	21	1	20	31.2 ± 2.46	29.0 ± 2.74	
	Berggreen (75)	TTH	Wait list	0–100 VAS	Treatment	20	1	19	28.0 ± 15.9	16.2 ± 11.8	
					Control	19	3	16	26.6 ± 12.6	24.9 ± 14.5	
	Ghanbari (74)	TTH	Routine medication	0–10 NPI^b^	Treatment	15	0	15	**58.0 ± 17.5**	**46.9 ± 24.9**	
					Control	15	0	15	**60.3 ± 9.9**	**63.6 ± 11.4**	
	Lawler (67)	MH	Wait list	0–5 pain scale	Treatment	24	1	23	No data^a^	No data^a^	
					Control	24	3	21	No data^a^	No data^a^	
	Ghanbari (76)	MH	Routine medication	0–5 pain scale^b^	Treatment	22	0	22	**66.8 ± 17.4**	**42.2 ± 11.6**	**39.0 ± 15**
					Control	22	0	22	**70.0 ± 14.2**	**55.8 ± 8.2**	**55.2 ± 14.8**

Duration of attacks	Moraska (64)	TTH	(1)Placebo	Hours × attack	Treatment	20	3	17	3.15 ± 0.43	2.65 ± 0.47	
			(2)WAIT list		Placebo (Detuned US)	21	2	19	2.86 ± 0.40	3.01 ± 0.44	
					Control (wait list)	21	1	20	3.02 ± 0.39	3.12 ± 0.43	
	Ghanbari (74)	TTH	Routine medication	Hours × attack	Treatment	15	0	15	6.42 ± 5.70	2.19 ± 2.28	
					Control	15	0	15	5.37 ± 3.47	5.02 ± 3.93	
	Ghanbari (76)	MH	Routine medication	Hours × attack	Treatment	22	0	22	35.59 ± 13.3	8.54 ± 3.76	8.95 ± 5.12
					Control	22	0	22	35.36 ± 8.85	19.22 ± 8.09	18.0 ± 9.27

*^a^Values indicated in bold have been normalized as attacks per month*.

*^b^Values indicated in bold have been normalized to a 0–100 numeric pain scale*.

*UC, usual care; RM, routine medication; US, ultrasound; TTH, tension type headache; MH, migraine; VAS, visual analog scale; NPI, numeric pain index*.

The summary of findings for each outcome in TTH and MH and the quality of assessment are reported in Table 5. The comparisons of interventions listed by outcome for both TTH and MH are reported in Appendix S2 in Supplementary Material.

**Table 5:** Summary of findings and quality of assessment.

Quality assessment							Summary of findings					Importance
			
							No. of patients		Effect	
		
No. of studies	Study design	Risk of bias	Inconsistency	Indirectness	Imprecision	Other considerations	Manual TrPs treatment	Other treatment	Relative (95% CI)	Absolute (95% CI)	Quality	
**Frequency (attacks/month)—TTH and MH pooled**												
6	Randomized trials	Serious^a^^,^^b^^,^^c^^,^^d^	Serious^e^	Not serious	Serious^f^	None	144	133	–	MD **3.05 lower** (4.11 lower to 2.00 lower)	⊕ΟΟΟ Very low	Critical

**Frequency (attacks/month)—TTH**												
4	Randomized trials	Serious^a^^,^^b^	Serious^e^	Not serious	Serious^f^	None	99	90	–	MD **3.50 lower**(4.91 lower to 2.09 lower)	⊕ΟΟΟ Very low	Critical

**Frequency (attacks/month)—MH**												
2	Randomized trials	Serious^a^	Not serious	Not serious	Serious^f^	None	45	43	–	MD **1.92 lower** (3.03 lower to 0.80 lower)	⊕⊕ΟΟ Low	Critical

**Intensity (0–100 scale)—TTH and MH pooled**												
6	Randomized trials	Very serious^a^^,^^b^^,^^c^^,^^d^	Very serious^g^	Not serious	Serious^f^	None	96	116	–	MD **12.93 lower** (18.70 lower to 7.16 lower)	⊕ΟΟΟ Very low	Important

**Intensity (0–100 scale)—TTH**												
4	Randomized trials	Very serious^a^^,^^d^^,^^f^^,^^h^	Very serious^g^	Not serious	Serious^f^	None	74	94	–	MD **12.83 lower** (19.49 lower to 6.17 lower)	⊕ΟΟΟ Very low	Important

**Intensity (0–100 scale)—MH**												
2^i^	Randomized trials	Very serious^a^^,^^d^	Not serious	Not serious	Very serious^f^^,^^j^	None	22	22	–	MD **13.60 lower** (19.54 lower to 7.66 lower)	⊕ΟΟΟ Very low	Important

**Duration (hours)—TTH and MH pooled**												
3	Randomized trials	Serious^a^	Very serious^g^	Not serious	Serious^f^	None	54	76	–	MD **1.69 lower** (2.93 lower to 0.46 lower)	⊕ΟΟΟ Very low	Important

**Duration (hours)—TTH**												
2	Randomized trials	Serious^a^	Serious^e^	Not serious	Serious^f^	None	32	54	–	MD **0.51 lower** (0.97 lower to 0.04 lower)	⊕ΟΟΟ Very low	Important

**Duration (hours)—MH**												
1	Randomized trials	Serious^a^	Not serious	Not serious	Very serious^f^^,^^j^	None	22	22	–	MD **10.68 lower** (14.41 lower to 6.95 lower)	⊕ΟΟΟ Very low	Important

*^a^Lack of blinding of participants and personnel*.

*^b^Lack of blinding of outcome assessment*.

*^c^Unclear allocation concealment*.

*^d^Selective reporting (reporting bias)*.

*^e^Moderate heterogeneity (50 < *I*^2^ < 75%)*.

*^f^Less than 400 subjects*.

*^g^High heterogeneity (*I*^2^ > 75%)*.

*^h^Incomplete outcome data (attrition bias)*.

*^i^One trial excluded from quantitative analysis due to reporting bias*.

*^j^Only one trial*.

*TTH, tension type headache; MH, migraine; TrPs, trigger points; MD, mean difference; CI, confidence interval*.

**Figure 4:**
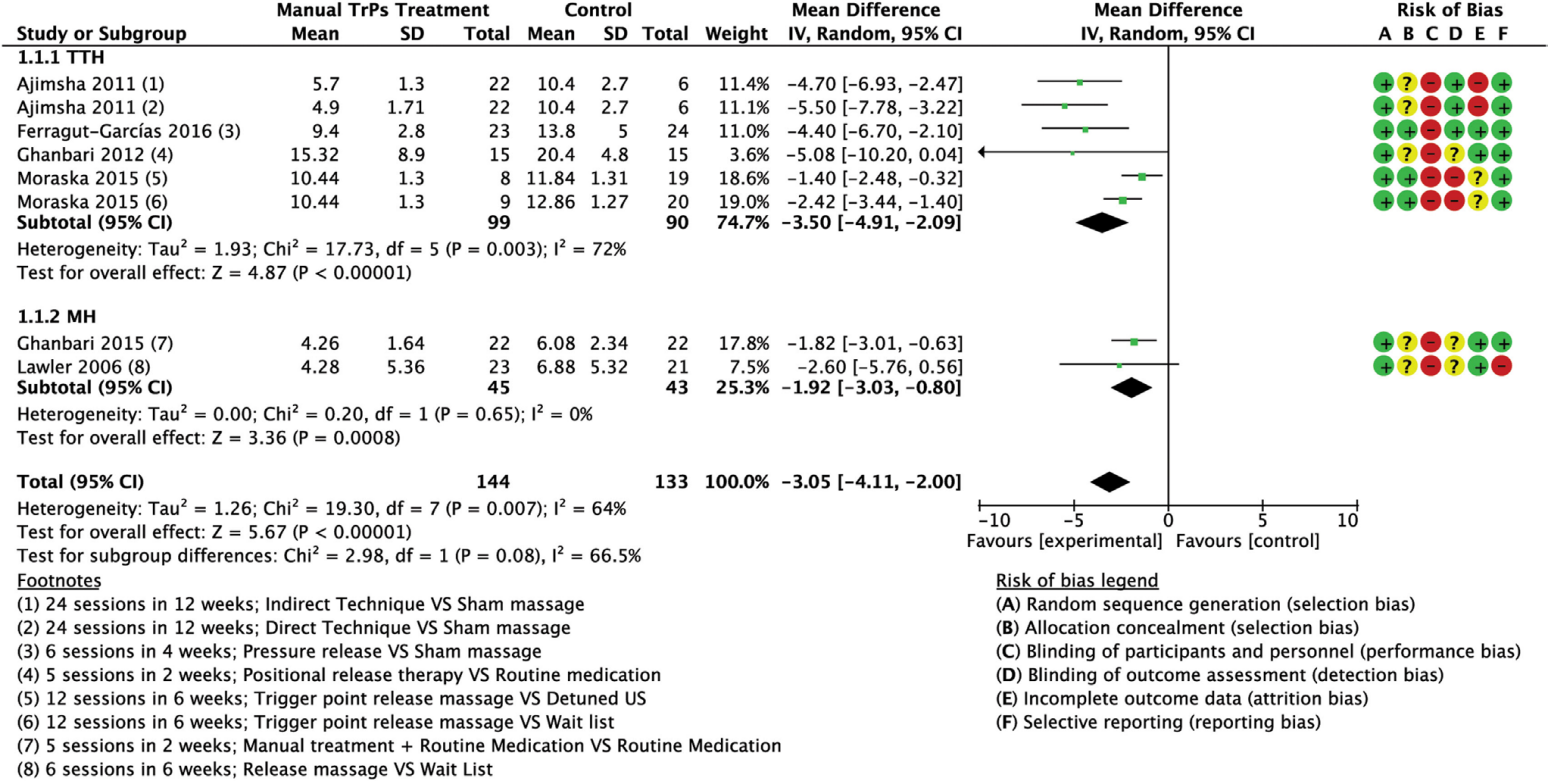
Forest plot of comparison for frequency (no. of attacks per month) compared with minimal active intervention in TTH (top) and MH (bottom). Abbreviations: TTH, tension-type headache; MH, Migraine; CI, confidence interval.

**Figure 5:**
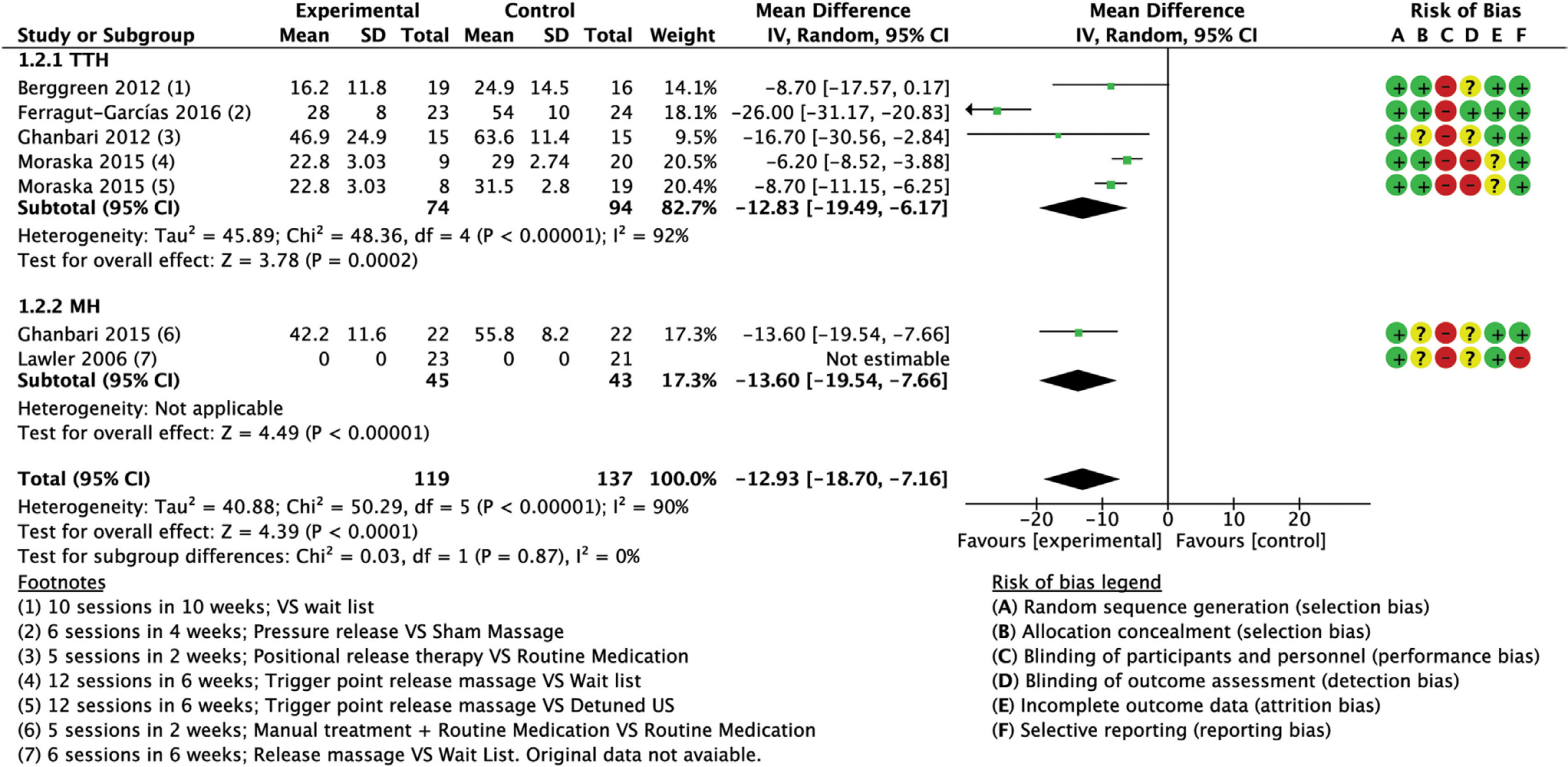
Forest plot of comparison for pain intensity (0–100 scale) compared with minimal active intervention in TTH (top) and MH (bottom). Abbreviations: TTH, tension-type headache; CI, confidence interval; MH, Migraine.

**Figure 6:**
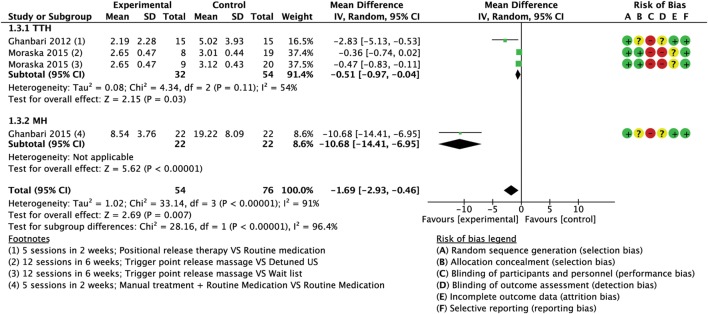
Forest plot of comparison for attacks duration (hours) compared with minimal active intervention in TTH (top) and MH (bottom). Abbreviations: TTH, tension-type headache; MH, Migraine; CI, confidence interval.

Meta-analyses of the pooled results with separated analyses of TTH and MH as subgroups are shown for frequency (Figure 4), intensity (Figure 5), and duration (Figure 6). Sensitivity analyses for RoB assessment were not conducted due to the insufficient numbers of studies in each pairwise comparison.

#### TrPs Manual Treatment vs Minimal Active Intervention on TTH and MH Combined

##### Frequency

A total of six trials (277 subjects) assessed the frequency of attacks as number of attacks per month; three trials had high RoB (64, 65, 67) and three had low RoB (60, 74, 76). The analysis of combined results indicated a statistically significant reduction in frequency of attacks per month after treatment favored the experimental group compared to control, with a mean reduction of 3.05 attacks/month (MD −3.05; 95% CI from −4.11 to −2.00; *P* < 0.01; *I*^2^ = 64%; see Figure 4).

##### Intensity

A total of six trials (256 subjects) assessed the intensity of pain using different scales (0–100) VAS (64, 75), 0–10 VAS/NPI (numeric pain index) (60, 74), 0–5 pain scale (76); only five trials were included into the quantitative analysis for this outcome due to missing original data from one study (67), although we tried to contact the authors. We found one trial with high RoB (64), while four trials had low RoB (60, 74–76). Analysis of combined results (referred to a normalized 0–100 scale) showed a significant reduction of intensity of attacks in the experimental group (MD −12.93; 95% CI from −18.70 to −7.16; *P* < 0.001; *I*^2^ = 90%; see Figure 5).

##### Duration

A total of three trials (130 subjects) assessed the duration of attacks; one had high RoB (64) and two had low RoB (74, 76). A mean reduction of 1.69 h per attack (MD −1.69; 95% CI from −2.93 to −0.46; *P* < 0.01; *I*^2^ = 91%; see Figure 6) favored the experimental group compared to control. A greater reduction was found in the trial concerning MH that compared the routine medication supported with the intervention versus routine medication alone with a mean reduction of 10.68 h per attacks (MD −10.68; 95% CI from −14.41 to −6.95; *P* < 0.001; see Figure 6).

#### Subgroup Analysis: Treatment Effects on TTH

##### Frequency

Four trials (189 subjects) of which two had high RoB (64, 65) and two had low risk (60, 74) which assessed the frequency of attacks. Analysis of combined results indicated a statistically significant reduction in frequency of attacks after treatment in the intervention group compared to control with a mean reduction of 3.50 attacks/month (MD −3.50; 95% CI from −4.91 to −2.09; *P* < 0.001; *I*^2^ 72%, see Figure 4).

##### Intensity

Four trials (168 subjects), three with low RoB (60, 74, 75) and one with high RoB (64) assessed intensity of pain using different scales [0–100 VAS (64, 75), 0–10 VAS/NPI (60, 74)]. The combined results (referred to a normalized 0–100 scale) indicated a statistically significant reduction in intensity of attacks after treatment in the intervention group compared to control group (CG) (MD −12.83; 95% CI from −19.49 to −6.17; *P* < 0.001; *I*^2^ = 92%, see Figure 5).

##### Duration

Two trials (86 subjects), one with high RoB (64) and one with low RoB (74) assessed the duration of attacks measured in hours. Analysis of combined results indicated a small reduction in duration of attacks after treatment in the intervention group compared CG with a mean reduction of 0.51 h per attacks (MD −0.51; 95% CI from −0.97 to −0.04; *P* = 0.03; *I*^2^ = 54%; see Figure 6).

#### Subgroup Analysis: Treatment Effects on MH

##### Frequency

Two trials (88 subjects), one with high RoB (67) and one with low RoB (76) assessed the frequency of attacks. A statistically significant reduction in frequency of attacks after treatment was observed in the intervention group with a mean reduction of 1.92 attacks/month (MD −1.92; 95% CI from −3.03 to −0.80; *P* < 0.001; *I*^2^ = 0%; see Figure 4).

##### Intensity

The same two trials (88 subjects) assessed the intensity of pain (0–5 pain scale). Lawler and Cameron (67) reported no significant group × time interaction effect revealed by the repeated measures ANOVA of changes from baseline to follow-up [*F*_(1, 42)_ = 0.82, ns; *d* = 0.11]; although we tried to contact the authors, we could not include these values in the meta-analysis as original data were unavailable (67). The results of the second trial (referred to a normalized 0–100 scale) indicated a statistically significant reduction in pain intensity (MD −13.60; 95% CI from −19.54 to −7.66; *P* < 0.001; see Figure 5).

##### Duration

Only one trial (44 subjects) with low RoB (76) reported a statistically significant reduction in duration of attacks after treatment in the intervention with a mean reduction of 10.68 h per attacks (MD −10.68; 95% CI from −14.41 to −6.95; *P* < 0.001; see Figure 6).

#### Additional Treatment Effects on TTH and MH

##### Number of Active TrPs

Number of Active TrPs in TTH, one RCT reported a significant decrease in the number of active TrPs in patients treated with ischemic compression (75).

##### Pressure Pain Threshold (PTT) and TrPs Sensitivity

Pressure pain threshold and TrPs sensitivity in TTH, two RCTs reported the increase of the PPT in treated muscles only in patients manually treated (*P* < 0.01) (60, 64); one RCT reported a significant reduction for sensitivity of TrPs only in the TG after intervention (*P* < 0.01) and at follow-up (*P* = 0.015) in TTH (74). The same results on TrPs sensitivity were reported in one RCT regarding MH (76).

##### Tablet Count and Medicine Consumption

Tablet count and medicine consumption in TTH, one RCT reported a significant reduction in tablet count after treatment phase (*P* < 0.01), but the result persisted only in manual TG after 2 weeks follow-up (*p* < 0.01) (74). A second study reported a not significant reduction of drug’s use only in the TG (*P* = 0.46) (75) while in a third study, no significant TG differences have been measured (64). In MH, one RCT reported a significant reduction in tablet count in both treatment (receiving manual PRT and medical therapy) and CG (receiving only medical therapy); differences between groups at various follow-up were always significant (*P* < 0.001) (76).

##### McGill Pain Questionnaire and Quality of Life (SF36)

In TTH, one study reported not significant differences between manual TG and control (75).

##### Headache Disability Inventory (HDI), Headache Impact Test-6 (HIT-6), and Perceived Clinical Change

In TTH, one study reported a significant decrease in HDI scores only in manual TG (*P* < 0.001) (64). Other two studies reported a significant group × time interaction in HIT-6 scores; Moraska et al. (64) detected effects over time for both manual treatment and placebo group (*P*’s < 0.01) but not in the CG (*P* = 0.52); Ferragut-Garcias et al. (60) detected effects over time for all manual TGs (*P*’s < 0.001) and CG (*P* < 0.05) compared to baseline. Regarding the perceived clinical change, greater pain reduction was observed for manual TG compared to placebo or CGs (*P* < 0.01) (64).

##### Quality and Quantity of Sleep and Stress and Coping (PSS)

Quality and quantity of sleep and stress and coping in MH the TG displayed a significant improvement of sleep quality, but not of the total amount of sleeping hours (67); in the same study, TG displayed no significant change in stress and coping levels during both treatment and at follow-up [state trait anxiety inventory scale (STAI) and perceived stress scale (PSS)]; otherwise a significant deterioration of these outcomes was found only in the CG (67).

##### Cervical ROM

In MH, one study reported a significant increase of cervical rotation in both experimental and CGs, but flexion, extension, and side bend increased only in experimental group after 4 months of follow-up (all *P*’s < 0.001) (76).

## Discussion

The present manuscript aimed at establishing the effectiveness of the TrPs manual treatment in reducing the frequency, intensity, and duration of attacks of primary headaches in adults. In order to explore this goal the authors’ agreement in performing articles selection and RoB assessment was moderate (Cohen’s *k* ranged from 0.69 to 0.72), supporting the methodological validity of the research.

The quality of the level of evidence ranged from low to very low in favor of manual TrPs treatment compared to minimal active intervention in the reduction of the frequency, intensity, and duration of the attacks in the patients with TTH and MH measured in the run-out period after intervention. Further findings having very low quality of the evidence were a greater reduction of frequency of attacks in patients with TTH and of the duration of attacks in the MH subgroup, while the intensity of attacks was similarly reduced in both subgroups.

The different extent of efficacy of the TrP treatment on the primary headaches considered either as separated entities or according to the continuum model needs to be substantiated in light of the most accepted hypotheses on the TrP induced pain. The integrated TrP hypothesis (33) proposed that a muscle overload causes ischemia and hypoxia in the muscle tissue leading to a cascade of biochemical events that finally ends in sarcomere contraction (e.g., a TrP) and produces nociceptive pain and tenderness of (pericranial) muscles. In contrast, the neuritis model (34, 78) hypothesized an inflammation of a peripheral nerve that produced a TrP as the result of ectopic impulses coming from the site of inflammation, while muscle pain and tenderness are described in terms of secondary hyperalgesia arising from the inflamed nerve. Considering primary headaches as separated entities, in case of TTH it has been proposed that the transformation from infrequent to chronic TTH is the result of the sensitization of the TNC (14, 43, 79) due to the presence of TrP according to the integrated TrP hypothesis. Thus, this mechanism is likely to explain the greater reduction of frequency and intensity of attacks for patients with TTH found in the present manuscript, but it is contradicted to some extent by the lack of reduction of duration of attacks as the de-sensitization of the TNC would also reduce the length of an attack likewise. In case of MH, it has been proposed according to the integrated TrP hypothesis that the nociceptive inputs arising from active TrPs may excite the TNC, activate the already sensitized trigemino-vascular system (16) and, consequently, promote a migraine attack (44). This mechanism may explain why frequency decreases to a little extent also in the MH group even though the reduction of intensity and duration of attacks seems paradoxical as the pain is driven by the trigemino-vascular system and not by the presence of TrPs which, constituting a precipitating factors, should influence only the frequency of the attacks. Furthermore, considering the vascular genesis proposed for TTH (15), the integrated TrP hypothesis is supported by the reduction of frequency of attacks with subtle effects on the reduction of duration of attacks, even though we should not expect a reduction of intensity of headache pain as previously described for MH.

The neuritis model better supports our results in terms of pooled groups and analyzed as subgroups. In fact, as MH may be the result of the central sensitization of the TNC due to inflammation of peripheral nerves it is possible that a TrPs treatment reduces frequency, intensity, and duration of MH attacks. Furthermore, supposing that the duration of attacks is driven by the central sensitization of the TNC according to the continuum model (30), the neuritis model may also explain why in TTH the duration of attacks is not reduced as TTH represents a mild form of migraine in which attacks are less intense and last less. However, the mechanisms of action of TrPs treatment with a neuritis model perspective remains unclear as TrPs are seen as the result of ectopic stimuli arising from the nerves and a link between muscular treatment and resolution of nerve inflammation has not been established yet apart from a reduction of mechanical forces on the nerves (80). Therefore, despite the proven effectiveness of the TrPs treatment on primary headaches, there is still the need to understand properly on which basis the manual TrPs treatment might work. For example, in one study (64) that delivered a sham treatment (detuned ultrasound) to the CG, an improvement was observed and this phenomenon pose several questions on which are the mechanisms underpinning the effectiveness of unblinded interventions, such as a placebo effect (81), as it may also activate cortical mechanisms of pain inhibition.

Our findings are in contrast with the result of a previous meta-analysis of Luedtke et al. (56) that reported no effect of physiotherapy (based mainly on exercises, modalities, relaxation techniques, and education) versus various comparators (any type of placebo intervention, any other active intervention as well as waiting list or standard care) in terms of intensity, frequency, and duration outcomes on primary headaches (56); however, authors included only one study adopting the TrP therapy, also present in the present manuscript (74), and did not consider primary headaches as an homogeneous group. It is, therefore, possible that the lack of specificity of treatments different from the TrPs therapy did not aim at the sensitization of the TNC and this may explain the different results. Finally, our results are consistent with two reviews reporting that a combination of massage therapy and exercise has the same efficacy of prophylactic treatment in patient with CTTH (52) and with MH (53).

At this time, we were unable to identify the most effective technique among those proposed as the treatments were delivered with a great variability in terms of techniques used (compression techniques, myofascial release, muscle energy, soft tissues, and positional release techniques) and their dosage (time of individual session, length of the treatment session, and the number of treatment per week); often the treatment protocols involved the use of more than a single technique. Furthermore, as the results were presented for MH and TTH merging episodic and chronic conditions, it was impossible to establish whether the effectiveness of TrPs manual treatment may differ among conditions with different frequency of attacks. Despite these considerations, both in TTH and MH, the muscles treated were mainly the sternocleidomastoideus, the upper trapezius, and the sub-occipitals and this could represent an important clinical indication for the operators.

Regarding pain reduction, although we have normalized the different scales to a single 0–100 scale, standardized unidimensional pain scales should be used in future studies to determine whether the obtained reduction has a clinical relevance and not merely a statistical significance.

Despite planned, we were not able to report on the additional negative effects of the manual treatment of TrPs as the included studies did not report them. The additional positive effects were reported sparsely in the retrieved studies, therefore, and they were grouped in three macro categories called medicine consumption, quality of life, and effects on TrPs. The evidences on reduction of medicine consumption were controversial: in patients with TTH one study reported a reduction (74, 76) and others no difference in the number of tablets taken (64, 75), while in MH the manual TrPs treatment may support the pharmacological treatment (75). The same doubts were present on the quality of life category, as there were no differences in questionnaires measuring general health (75) in patient with TTH and anxiety and stress in patients with MH (67). An effect was found for questionnaires like the HDI and the HIT-6 (60, 64). Furthermore, despite a similar amount of sleeping hours, the quality of sleep improved in patients with MH (67). For the category effects on TrPs, a reduction of the number of active TrPs (75) and an increased pressure pain threshold were found in the muscles manually treated (64, 74).

Our findings must be interpreted with caution for the weakness of the level of the evidence mainly due to the high RoB within studies and imprecision in results. Moreover, as the majority of the included studies had short-term follow-up, the long-term effect of TrPs manual treatment still needs to be established.

Performance and detection bias mainly constituted the judgment of high RoB across our trials downgrading the level of evidence. Performance bias refers to systematic differences between groups in the care that is provided, or in exposure to factors other than the interventions of interest. Detection bias refers to systematic differences between groups in how outcomes are determined (82). Blinding may reduce the risk that knowledge of which intervention was received, rather than the intervention itself, affects outcomes: it is a cornerstone of treatment evaluation (83). Lack of blinding in RCTs has been shown to be associated with more exaggerated estimated intervention effects (84, 85). As expected, blinding of participants and providers was not present in all of our studies. In fact, blinding is more difficult to obtain in trials assessing non-pharmacological treatments, such as manual therapy. Therefore, researchers and readers must be aware of existing methods of blinding to be able to appraise the feasibility of blinding in a trial (83). For example, in manual therapy, patients could be blind to the active placebo therapy (83). However, if blinding is not possible researchers could standardize the treatment of the groups (apart from the intervention), consider an expertise-based trial design, use objective, and reliable outcomes if possible, and consider duplicate assessment (86). Nevertheless, when performance bias could not be avoided, at least blinding of assessors must be performed to ensure unbiased ascertainment of outcomes (87), mostly in subjective outcomes (84). Furthermore, considering the chronicity of primary headaches, the use of single case research design (SCR) to demonstrate treatment efficacy may also have a role (88). SCR has been considered as a possible method for the scientific evaluation of manual therapies (89) and several reports argue for this (90–92).

The second important reason for downgrading the level of evidence was imprecision in results mainly due to an unwarranted paucity of participants included in the comparisons (mean of 45 subjects) that avoid to reach the optimal information size. Considering the high prevalence of headache and the largely adopted manual treatments, physical therapists and researchers should demonstrate that manual therapy represents a valid option (for example, no risk of toxicity or overuse of medications) in the spectrum of headache therapies. Only if they invest more efforts to enhance powered and well-designed RCTs, the interventions can be universally accepted by the evidence-based medicine. Therefore, we call for a launch of further well-designed trials. Particularly, we encourage clinicians, researchers, and all stakeholders to promote multicenter trials focused in manual therapy treatment for TTH and/or MG. Multicenter trials should be based on a powered sample size calculation needed to reach the clinical relevance of the manual treatment versus the minimal intervention. The RoB should be minimized at least through the blinding of assessment. Moreover, a trial sequential analysis could be proposed in order to aim at the firm evidence, confirming or confuting our preliminary results.

## Limitations

The present review has some limitations that need to be addressed. Because we did not attempt to identify unpublished RCTs and our inclusion criteria were limited to only three languages, a publication bias could have occurred. The high variability of the delivered treatments prevented us from the identification of the most effective technique among those proposed. Even if epidemiological studies have determined that women are more likely to suffer from TTH and that female gender constitutes a risk factor for this disease (93), the higher prevalence of women in the TTH subgroup could make the results less applicable to the general population.

## Conclusion

There was very low evidence that manual TrPs treatment of the head and neck muscles may constitute a useful treatment to reduce frequency, intensity, and duration of attack in patients with TTH and MH. The included studies did not report any additional negative effects, while positive effects regarding reduction of medicine consumption were controversial.

## Author Contributions

FML prepared the draft, planned the overall design, performed the search and quality/methodological assessment of the included studies. GT had the original idea of the study, planned the design, prepared the draft, and revised the drafted manuscript. ZM performed the search and quality/methodological assessment of the included studies. GS revised the methodological part and the synthesis of results. TM revised the final manuscript. All five authors have read and approved the final manuscript before submission.

## Conflict of Interest Statement

The authors declare that the research was conducted in the absence of any commercial or financial relationships that could be construed as a potential conflict of interest.

## Abbreviations

CTTH, chronic tension type headache; ETTH, episodic tension type headache; FETTH, frequent episodic tension type headache; MH, migraine; CH, cluster headache; TG, treatment group; CG, control group; PG, placebo group; MCIDs, minimal clinical important difference; PIR, post isometric relaxation; CRAC, contract relax antagonist contraction; DT/IDT-MFR, direct/indirect myofascial release; SSTM, specific soft tissue mobilization; PRT, positional release therapy; TNC, trigeminal nucleus caudalis.
