# Supplementary material for: Effectiveness of Trigger Point Manual Treatment on the Frequency, Intensity, and Duration of Attacks in Primary Headaches: A Systematic Review and Meta-Analysis of Randomized Controlled Trials
Source: Front Neurol. 2018 Apr 24;9:254. doi: 10.3389/fneur.2018.00254 (PMC5928320; doi:10.3389/fneur.2018.00254)
Supplement: Supplementary file 1 [file Data_Sheet_1.docx]

Appendix 1: search strategy and entry terms (no filters applied; last search 17 Nov 2017)

| # | MEDLINE | Records |
| --- | --- | --- |
| 1 | ("Manual therapy" OR "trigger point" OR "myofascial trigger point" OR "Trigger Points"[Mesh] OR "Musculoskeletal Manipulations"[Mesh] OR "Musculoskeletal manipulations") AND ("Tension-Type Headache" OR "Cluster Headache" OR "Migraine Disorders"[Mesh] OR "migraine" OR "headache"[MeSH Terms] OR "Headache Disorders, Primary"[Mesh] OR "primary headache") | 408 |
| # | COCHRANE DATABASE | Records |
| 2 | ("Manual therapy" OR "trigger point" OR “myofascial trigger point” OR “Musculoskeletal manipulations” OR "Myofascial pain syndrome") AND ("Tension-Type Headache" OR "Cluster Headache" OR “migraine” OR "Migraine with Aura" OR "headache" OR “primary headache”) | 139 |
| # | WEB OF SCIENCE DATABASE | Records |
| 3 | ("Manual therapy" OR "trigger point" OR “myofascial trigger point” OR “Musculoskeletal manipulations” OR "Myofascial pain syndrome") AND ("Tension-Type Headache" OR "Cluster Headache" OR “migraine” OR "Migraine with Aura" OR "headache" OR “primary headache”) | 334 |
| # | PEDRO (no filters applied) | Records |
| 4 | headache AND "trigger point" | 14 |
| 5 | migraine AND "trigger point" | 2 |
| 6 | primary headache AND manual therapy | 12 |
| 7 | tension-type headache AND “trigger point” | 5 |

Appendix 2: comparisons of interventions listed by outcome. Note: TTH: Tension Type Headache; MH: Migraine; MD: Mean Difference; CI: Confidence Interval; RCT: Randomized Controlled Trial.

| Outcome and Subgroup | RCT | Participants | Statistical Method | Effect Estimate |
| --- | --- | --- | --- | --- |
| 1.1 Frequency  (attacks/month) | 6 | 277 | MD (IV, Random, 95% CI) | -3.05 [-4.11, -2.00] |
| 1.1.1 TTH | 4 | 189 | MD (IV, Random, 95% CI) | -3.50 [-4.91, -2.09] |
| 1.1.2 MH | 2 | 88 | MD (IV, Random, 95% CI) | -1.92 [-3.03, -0.80] |
| 1.2 Pain Intensity  (0-100 pain scale) | 6 | 256 | MD (IV, Random, 95% CI) | -12.93 [-18.70, -7.16] |
| 1.2.1 TTH | 4 | 168 | MD (IV, Random, 95% CI) | -12.83 [-19.49, -6.17] |
| 1.2.2 MH | 2 | 88 | MD (IV, Random, 95% CI) | -13.60 [-19.54, -7.66] |
| 1.3 Duration  (hours/attack) | 3 | 130 | MD (IV, Random, 95% CI) | -1.69 [-2.93, -0.46] |
| 1.3.1 TTH | 2 | 86 | MD (IV, Random, 95% CI) | -0.51 [-0.97, -0.04] |
| 1.3.2 MH | 1 | 44 | MD (IV, Random, 95% CI) | -10.68 [-14.41, -6.95] |
